# UTAP: User-friendly Transcriptome Analysis Pipeline

**DOI:** 10.1186/s12859-019-2728-2

**Published:** 2019-03-25

**Authors:** Refael Kohen, Jonathan Barlev, Gil Hornung, Gil Stelzer, Ester Feldmesser, Kiril Kogan, Marilyn Safran, Dena Leshkowitz

**Affiliations:** 1 Bioinformatics Unit, Department of Life Sciences Core Facilities, Weizmann Institute of Science, 76100 Rehovot, Israel; 2 The Mantoux Bioinformatics institute of the Nancy and Stephen Grand Israel National Center for Personalized Medicine, Department of Life Sciences Core Facilities, Weizmann Institute of Science, 76100 Rehovot, Israel

**Keywords:** NGS, Transcriptome, RNA-Seq, Sequence analysis pipeline, Bioinformatics workflow, Differentially expressed genes, Genome mapping, Bulk MARS-Seq, UMI (unique molecular identifier), Gene expression profile, Normalization

## Abstract

**Background:**

RNA-Seq technology is routinely used to characterize the transcriptome, and to detect gene expression differences among cell types, genotypes and conditions. Advances in short-read sequencing instruments such as Illumina Next-Seq have yielded easy-to-operate machines, with high throughput, at a lower price per base. However, processing this data requires bioinformatics expertise to tailor and execute specific solutions for each type of library preparation.

**Results:**

In order to enable fast and user-friendly data analysis, we developed an intuitive and scalable transcriptome pipeline that executes the full process, starting from cDNA sequences derived by RNA-Seq [Nat Rev Genet 10:57-63, 2009] and bulk MARS-Seq [Science 343:776-779, 2014] and ending with sets of differentially expressed genes. Output files are placed in structured folders, and results summaries are provided in rich and comprehensive reports, containing dozens of plots, tables and links.

**Conclusion:**

Our **User**-friendly **T**ranscriptome **A**nalysis **P**ipeline (UTAP) is an open source, web-based intuitive platform available to the biomedical research community, enabling researchers to efficiently and accurately analyse transcriptome sequence data.

## Background

Next-generation sequencing (NGS) technologies are the most advanced molecular tools currently available to interrogate the complexities of the transcriptome[1, 5], with proven efficient and cost-effective mechanisms for studying gene expression and reliably predicting differential gene expression [6]. Many methods for preparing the libraries have emerged, including Poly A or RiboZero for mRNA enrichment, complete transcript sequencing, strand-specific sequencing [2] and 3′ UTR sequencing [7]. In addition, in cases of initial low RNA levels, unique molecular identifiers (UMIs) are often incorporated in order to label individual cDNA molecules with a random nucleotide sequence before amplification. Advances in short-read sequencing instruments have yielded easy-to-operate machines, with high throughput, at a low price per base.

**Fig. 1:**
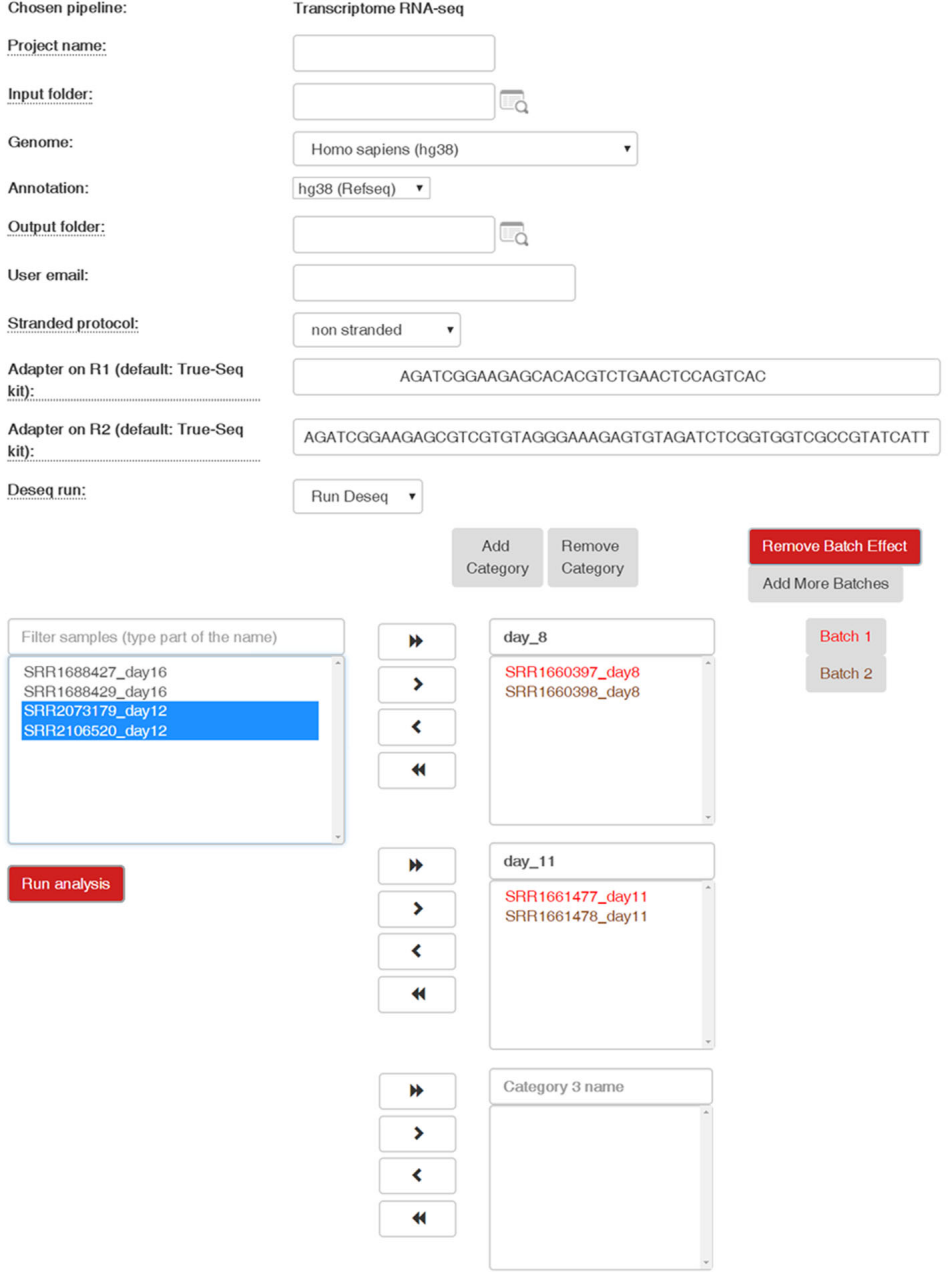
An example of a page in the pipeline’s Web Graphical Interface. Demonstrates the information required from the user in order to run the pipeline

The massive amount of data created by NGS requires bioinformatics expertise to tailor specific solutions for each type of library preparation. Implementing the solutions typically requires scripting and running commands in the *Linux* environment. An example of such protocols can be seen at [8]. To address this challenge and simplify the analysis, we developed a transcriptome pipeline, with an intuitive user interface (Fig. 1; results in supplementary materials; demonstration).

## Implementation

### Workflow

The UTAP system is composed of a *Snakemake* [9] workflow system backend, and *Python* (v2.7) and a *Django* (v1.11) - based web user interface (WUI) through which users can run analyses.

*Snakemake* bundles in-house scripts (written in *Python* and *R*) and public bioinformatics tools for completing stepwise processes. Sequence quality control is assessed by *FastQC* (v0.11.7), read-genome mapping by *STAR* [10] (v2.5.2b), gene count calculation by either *STAR* or *HTSeq* [11] (0.9.1) along with our specialized scripts for UMI counting. *SAM* and *BAM* file manipulation is accomplished by *Samtool*s [12] (v1.6), and gene body coverage plotting is performed by *ngsplot* [13] (v2.61). Differentially expressed genes (DEG) detection and count normalization analysis are performed by *DESeq2* [14] (1.18.1). The R package *fdrtool* [15] (1.2.15) is used to adjust *p* values when UTAP deduces that the raw *p*-value distribution is biased. The *sva* [16] (3.26.0) R package is used for batch correction of the counts when batch adjustments are required.

### Web Interface

To increase usability, thereby broadening the potential audience of UTAP, the WUI was planned to be intuitive. Researchers select a pipeline type (demultiplexing or transcriptome), provide the Illumina sequence data (bcl or fastq files), and choose the relevant genome and its annotation source (GENCODE or RefSeq). When running DESeq2, samples should be grouped by category and can be assigned to batches, using a select and drag approach (Fig. 1; supplementary information; demonstration). Batches are sub-groups of measurements that might have qualitatively different behaviour across conditions, and are unrelated to the biological or scientific variables in the study.

### Packaging

UTAP is available as a Docker image, which can run locally on one server, or integrated into LSF (Platform Load Sharing Facility, IBM) or PBS professional (OpenPBS; http://www.pbspro.org/) HTC (High-throughput computing) clusters.

### Customization

We chose the various pipeline parameters based on our rich experience in transcriptome analysis. This works very well for users who are not deeply familiar with bioinformatics software, and who prefer to quickly benefit from these choices without having to delve into the pipeline’s architecture. On the other hand, many research groups have their own particular preferences, and can achieve system-wide and/or run-specific flexibility by making adjustments to the parameters or code (Snakefile, R scripts) as described in the guide.

## Results

Our **U**ser-friendly **T**ranscriptome **A**nalysis **P**ipeline (UTAP) requires minimal user intervention. After providing the information described above (see demonstration), all steps required per library type are automatically executed. Upon completion, the system produces a rich and structured report as output. The transcriptome pipeline is designed for stranded or non-stranded TruSeq libraries, or, alternatively, for bulk RNA 3′ UTR MARS-Seq samples.

**Fig. 2:**
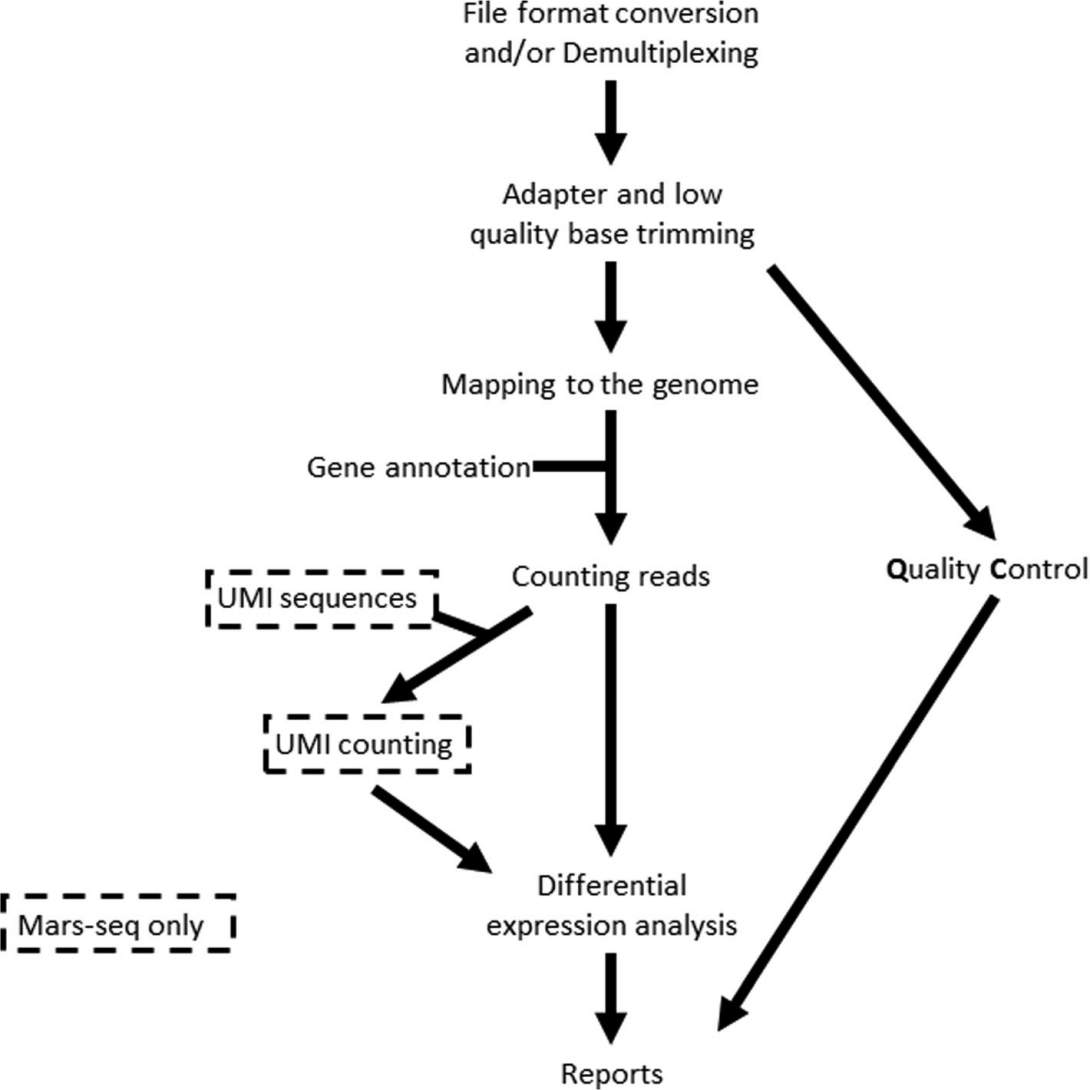
Flow of analysis step performed by the UTAP pipeline. *Note that steps that take place only in the MARS-Seq pipeline are shown within broken-line rectangles*

The pipeline runs the following steps (see Fig. 2 and examples in supplementary materials): demultiplexing, adapter and low-quality trimming, quality checks, mapping to a genome, gene quantification, UMI counting (if required), normalization, and detection of statistically significant differentially expressed genes (DEG) for pairwise comparisons of user-defined categories. Once a run has been completed, the user can redefine the samples and categories and rerun only DESeq2. If batches are defined, DESeq2 analyses take them into account.

**Fig. 3:**
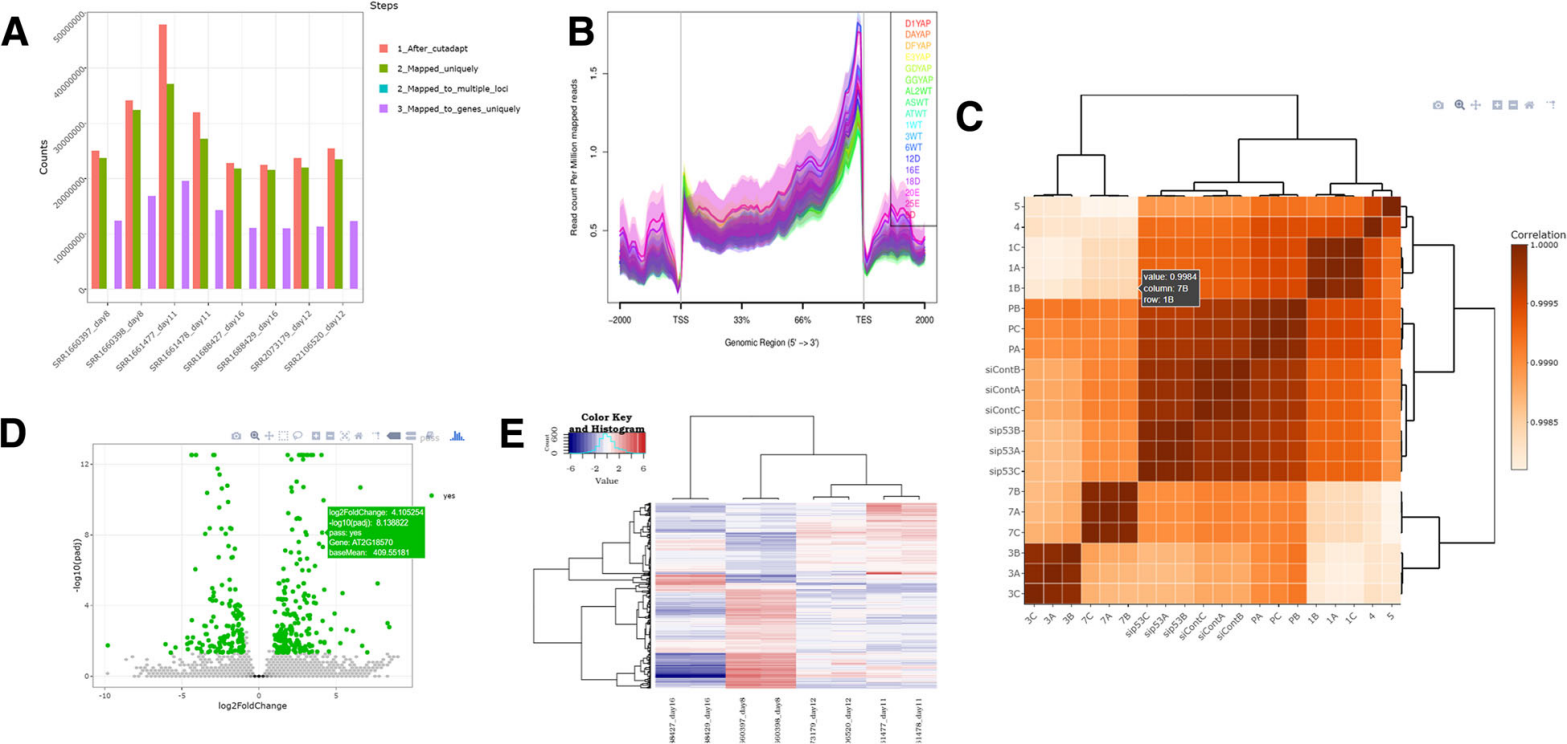
Selection of plots produced in a UTAP report. **a** Histogram with the number of reads for each sample in the various pipeline steps. **b** Sequence coverage on and near gene regions using *ngs.plot* [13] **c**. Heatmap of Pearson correlation between samples according to gene expression values. **d**. Scatter plot of significance (y axis) versus fold-change (x axis). **e** Hierarchical clustering heatmap of differentially expressed genes. Plots D and E are created when DESeq2 analysis is executed

The comprehensive report (see Fig. 3 and examples in supplementary materials) contains dozens of figures for visual inspection, including statistical information, enabling one to explore the efficiency of the process. The figures contain details covering the number of reads per sample in the various steps of the process, the amount of similarity between the samples, and more. In addition, the report contains tables with information on the DEG in each category (up/down) as well as links to gene annotation at *GeneCards* [17] and submitting gene sets for pathway analysis on *Intermine* [18]. The report closes with a description of the databases, tools and parameters used, and links to additional results. All pipeline outputs, such as trimmed fastq files, mapped and indexed bam files, matrices of raw, normalized counts and statistical DEG values, are available in structured folders. R scripts containing code for plots and statistics and logs are also included, thus packaging the analysis into a reproducible format.

The pipeline is scalable, utilizing the full power of the server or cluster. The Docker image has been tested on LSF and OpenPBS clusters. The scalability allows for fast processing of the data. When the pipeline runs in parallel on each sample with 20 threads per sample, the run time is ~ 1 h for MARS-Seq analysis and ~ 2.5 h for RNA-Seq analysis.

A collection of features that significantly differentiates UTAP from previously reported pipelines and platforms [19–25] is presented in Table 1. Specifically, the other platforms either lack a friendly graphical user interface, and/or are not scalable, and/or have complex installations, and/or do not provide predefined pipelines, and/or do not provide meticulous ways to detect differentially expressed genes, and/or do not have structured outputs. All of the other systems create reproducible results, but lack analysis for bulk MARS-Seq, and do not automatically create summaries via comprehensive reports.

**Table 1:** Comparison of Transcriptome Analysis Pipelines

Tool/platform	Graphical user interface (GUI)	Workflow	DEG detection	Scalable (cluster)	Hosting	Installation	Reproducible runs	Automatic comprehensive report with statistics	Structured output folders	Bulk MARS-Seq	NGS tools other than for DE	Ref
Chipster	Yes	user defined	Yes	Yes	Local, Remote server and cloud	Medium (requires virtualization sw)	Yes	No	No	No	Yes	19
RNACocktail	No	predefined	Yes	No	Local	Easy (Docker)	Yes	No	No	No	Yes	20
hppRNA-a	No	predefined	Yes	Yes	Local	Medium (Installation script)	Yes	No	Yes	No	Yes	21
aRNApipe	No	predefined	No	Yes	Local	Medium	Yes	Partial (no DEG)	Yes	No	Yes	22
Galaxy	Yes	user defined	Yes	Yes	Local, Remote server and cloud	Complex	Yes	No	No	No	Yes	23
Illumina BaseSpace	Yes	predefined	Yes	Yes	Remote (requires a fee)	NA	Yes	Partial	Yes	No	Yes	24
docker4seq	Yes	predefined	Yes	Yes	Local	Easy (Docker)	Yes	No	Yes	No	Yes	25
UTAP	Yes	predefined	Yes	Yes	Local	Easy (Docker)	Yes	Yes	Yes	Yes	No	NA

Our future plans include improving customization by providing options to modify parameters via the web interface, adding NGS pipelines such as small RNAs, ChIP-Seq, ATAC-Seq, Ribo-Seq, SNP detection in RNA-Seq and single-cell RNA-Seq, and adapting the pipeline to run on other types of computing clusters and in the cloud.

## Conclusions

UTAP is an open source, web-based intuitive, scalable and comprehensive platform available to the biomedical research community. It executes an efficient and accurate analysis of transcriptome sequence data, producing sets of differentially expressed genes and sophisticated reports, and requiring minimal user expertise.

## Availability and requirements

**Project name**: UTAP: User-friendly Transcriptome Analysis.

**Pipeline Installation manual**: https://utap.readthedocs.io

**Operating system(s)**: Linux.

**Programming language**: Python v2.7, R.

**Other requirements**: Docker v1.7, miniconda v2.

The pipeline consumes ~40GB RAM. The required disk space for the output files is ~1GB per sample for MARS-Seq analysis and ~6GB per sample for RNA-Seq analysis. In addition, ~135GB are required for storage of the genome files.

**License**: GNU GPL version 3.

**Any restrictions to use by non-academics**: License needed for commercial use.

## Abbreviations

BAM: Binary alignment map
DEG: Differentially expressed genes
GB: Gigabyte
NGS: Next generation sequencing
RAM: Random access memory
SAM: Sequence alignment map
SNP: Single nucleotide polymorphism
UMI: Unique molecular identifier
WUI: Web user interface
